# The role of virus infections in Sjögren’s syndrome

**DOI:** 10.3389/fimmu.2022.823659

**Published:** 2022-09-06

**Authors:** Maria Maslinska, Kinga Kostyra-Grabczak

**Affiliations:** National Institute of Geriatrics, Rheumatology and Rehabilitation, Warsaw, Poland

**Keywords:** Sjögren’s syndrome, herpesviruses, retroviruses, hepatitis viruses, autoimmunity, viral infections

## Abstract

Primary Sjögren’s syndrome (pSS) is an autoimmune disease with a clinical picture of not only mainly exocrine gland involvement, with dryness symptoms, but also internal organ and systems involvement. The epithelial damage and releasing of antigens, which, in some circumstances, become autoantigens, underlay the pathogenesis of pSS. The activation of autoimmune processes in pSS leads to the hyperactivation of B cells with autoantibody production and other immunological phenomena such as hypergammaglobulinemia, production of cryoglobulins, or formation of extra-nodal lymphoid tissue. Among the risk factors for the development of this disease are viral infections, which themselves can activate autoimmune reactions and influence the host’s immune response. It is known that viruses, through various mechanisms, can influence the immune system and initiate autoimmune reactions. These mechanisms include molecular mimicry, bystander activation, production of superantigens—proteins encoded by viruses—or a programming to produce viral cytokines similar to host cytokines such as, e.g., interleukin-10. Of particular importance for pSS are viruses which not only, as expected, activate the interferon pathway but also play a particular role, directly or indirectly, in B cell activation or present tropism to organs also targeted in the course of pSS. This article is an attempt to present the current knowledge of the influence specific viruses have on the development and course of pSS.

## Introduction

In many rheumatic diseases, the influence of viral infections is considered as a potential triggering factor for the activation of an autoimmune process. Several viruses are suggested to have an effect on autoimmunity—among them are Epstein–Barr virus (EBV) and human herpes virus-6 (HHV-6), hepatitis C (HCV) and B (HBV) viruses, enteroviruses, influenza A virus, human immunodeficiency virus (HIV), or viruses of Flaviviridae family (e.g., Zika and dengue viruses) (1). In the case of some viruses, the mechanism of their influence on autoimmunity is uncertain as the autoimmune reaction is triggered by the reactivation of a virus that previously remained in a latent state (e.g., polyomavirus JC virus). Some of the viruses involved in autoimmune processes infect exclusively humans, with a virus targeting only specific host cells (e.g., B cells)—e.g., EBV. Environmental factors, including viral infections, lead to the development of the autoimmune reaction by essentially causing a breakdown of autotolerance, which triggers the production of autoantibodies and the development of specific clinical phenomena. Viruses influence the immunity through mechanisms of molecular mimicry, generation of superantigens, apoptosis, necrosis, clearance deficiency, and bystander activation (1, 2).

Primary Sjögren’s syndrome (pSS), also known as “autoimmune epithelitis”, is a model autoimmune disease, in which damage to the epithelial barrier, release of autoantigens, and the development of a cascade of autoimmune reactions constitute the core of the problem. The epithelial damage and the activation of B cells are essential for the activation of the immune system in pSS. These elements and the whole cascade of autoimmune phenomena can be stimulated at various stages by environmental factors, including viral infections. The aim of this article is to discuss the influence of viral infections on the development and course of pSS.

## Primary Sjögren’s syndrome

Primary Sjögren’s syndrome is an autoimmune disease with inflammatory infiltration, with mononuclear cells affecting primarily the exocrine glands, epithelium damage, and hyperreactivity of B cells with autoantibodies against ribonucleoproteins: anti-SSA/Ro and anti-SSB/La production (3, 4).

The main clinical feature of pSS is mucosal dryness of the mouth, eyes, vagina, and bronchial tree, which occurs due to the involvement of the exocrine glands. Dryness symptoms may occur late in the course of the disease, leading to the undetected development of pSS for a long period of time, with the disease expressed initially through chronic fatigue, joint pain, or formation of stones in the kidneys, gallbladder, or salivary glands. Primary Sjögren’s syndrome, as a systemic disease, may progress with the involvement of internal organs manifesting itself as an interstitial lung disease, autoimmune cholangitis, hepatitis, vasculitis in the form of skin lesions (livedo reticularis and palpable purpura), and peripheral or central nervous system dysfunction. This often leads to the patient being referred to various specialists before the pSS diagnosis is eventually established (5). An active HCV infection (often asymptomatic in the area of hepatic lesions) may take the form suggesting a systemic disease—including pSS. Therefore, exclusion of HCV infection is an indispensable part of the differential diagnosis of pSS, as reflected in the pSS diagnostic criteria (5).

The main histopathologic feature of pSS is the presence of mononuclear cell infiltrations in the salivary glands and often in other internal organs as well. Infiltrations include dendritic cells (DC), CD 4+, and plasma cells (6). Cell clusters can form germinal centers (GC) and ectopic lymphoid tissue, the emergence of which increases the risk of developing lymphomas (mainly B cell) (6).

The pathogenesis of pSS is thought to consist in the damage of the epithelium and the release of antigens (7), with pSS being sometimes described as “autoimmune epithelitis”. The autoantigens Ro52, Ro60, and La (48 kD) are associated with pSS, but the mechanism of the breakdown of the immune tolerance remains unclear. Apoptosis is suggested to cause the presentation of these autoantigens to the immune system (8). Subsequently, the innate immune system response starts. The activation of Toll-like receptors (TLR) and the stimulation of DC and macrophages trigger, in turn, the release of interferons (IFNs), the stimulation of T cells (especially CD 4+), and the production of B cell-stimulating factors—such as BAFF and a proliferation-inducing ligand (APRIL) (7). The hyperactivation of B cells is key to this disease, as it leads to hypergammaglobulinemia and the production of anti-ribonucleoprotein antibodies (anti-SSA/Ro and SSB/La antibodies). The anti-SSA/Ro antibodies recognize two cellular proteins, which have a molecular weight of 52 and 60 kD and are therefore named anti-Ro52 and anti-Ro60, respectively (8, 9). Those autoantibodies do not cross-react with each other. They are also located in various cell regions: Ro60 in the nucleus and nucleolus and Ro52 in the cytoplasm. They are also encoded by two different genes: located on chromosome 19 for Ro60 and on chromosome 11 for Ro52 (10). It turned out that Ro60 exists as a small cytoplasmic conserved protein which binds RNA (hY-RNA) (11). Ro52 was discovered (in 1988) to be a part of the SSA complex. Later, it was identified as an E3 ubiquitin ligase (12) and a protein belonging to the tripartite motif (TRIM) protein family (13). Anti-SSA/Ro antibodies, particularly anti-Ro (52 kD), are detected in several autoimmune diseases, especially in SLE, RA, idiopathic inflammatory myopathy, dermatomyositis, and systemic sclerosis (14). Importantly, Ro52 is a protein, the production of which is induced by viral infections through the stimulation of TLRs and of the interferon I pathway (IFN I) (15). It is suggested that anti-Ro60 is more specific for pSS than anti-Ro52 (16). The SSB/La protein is a transcription–termination factor for RNA polymerase III. The anti-SSB/La antibodies commonly occur along with anti-SSA/Ro antibodies; they are present in some pSS patients and in SLE patients. Their presence is associated with neutropenia and cryoglobulinemia. In maternal SLE the presence of anti-SSB/La and anti-SSA/Ro antibodies is associated with disorders of the heart conduction system during fetal development and, after birth, in neonatal lupus (17, 18).

The tropism of some viruses to exocrine glands, such as salivary glands (e.g., EBV) may cause the development of dryness symptoms in the course of the infection, requiring the differentiation of such phenomena from pSS. At the same time, the same viruses may also contribute to the development of pSS.

Recognized genetic risk factors for the development of pSS include the presence of certain human leukocyte antigen (HLA) system genotypes (HLA-B8, HLA-DW3, HLA-DR3, and DRw52) and a polymorphism of genes for interferon regulatory factor 5 (IRF5) and signal transducer and activator of transcription 4 (STAT4) [which are both gene activators of the immune response of the type I of interferon (IFN) system], CXCR5 (cell surface protein found in antibody-producing B cells), TNIP1 (binding partner for TNFAIP3—protein which plays a role in limiting inflammation), IL12A (part of this protein activates T cells and natural killer cells), and BLK (tyrosine protein kinase, which activates B cells) (19–22). The epigenetic phenomena (the influence of environmental factors on human genes through the mechanisms of DNA methylation and histone modification of non-coding RNA) (23), which result in the modification of the interferon pathway, are also considered as risk factors for pSS development. Such changes may occur in pSS in peripheral blood cells, serum, and saliva (24). These processes are reversible and represent a potential target for new therapies being developed (25).

The estrogen imbalance is considered as a hormonal risk factor in pSS. In genetically predisposed individuals, the decrease or lack of estrogens may lead to autoimmunity development and dry eye syndrome and may result in a more severe course of the disease—which was confirmed both in animal studies and in clinical observations (26, 27).

## Principles of primary Sjögren’s syndrome diagnosis

The current classification criteria for pSS, employed in the diagnosis of this disease, were published in 2016 (5). The features that are most important for pSS diagnosis include confirmation of dryness of eyes/mouth, presence of the SS-A/Ro antibodies, and presence of typical infiltrates consisting of mononuclear cells in the assessment of histopathological material from labial minor salivary gland biopsy (MSGB). The latter must meet the so-called focus score (at least one focus present with a minimum of 50 mononuclear cells in a 4-mm^2^ biopsy section). The patient must also be checked by a clinician for the presence of exclusion criteria, which include irradiation of the head and neck, active HCV infection, diagnosis of IgG4-related disease (IgG-RD), and presence of lymphoma, making the diagnosis impossible. In **Table 1**, the scoring system for the current main pSS classification criteria is presented. According to the classification criteria, pSS diagnosis requires a total score of ≥4 points (5, 28).

**Table 1:** Main items and scoring in the recent pSS classification criteria (acc. 5).

Parameter	Scoring (points)	Comment
Schirmer’s test ≤5 mm/5 min in at least one eye or ocular staining score (OSS) ≥5 or van Bijsterveld’s score ≥4 ) in at least one eye	1 1 1	Without using artificial saliva Using lissamine green and fluorescein staining Lissamine green or Bengal rose staining
Unstimulated salivary flow ≤0.1 mL/min	1	Without using artificial saliva
Presence of anti-SSA/Ro antibodies	3	Without division into Ro52 and Ro60
Focus score ≥1 foci/4 mm2	3	Important representative

## Viral infections and pSS—potential mechanisms of virus influence on pSS development

Infections, especially viral ones, play a crucial role in pSS pathophysiology. Several mechanisms found to be important for the effect of viruses on pSS are presented below.

### Molecular mimicry

Molecular mimicry is indicated as the mechanism of adaptation by a pathogen to avoid immune detection by mimicking a host antigen for which immune tolerance exists. It facilitates virus penetration into the host’s cells, utilizing the host’s immune tolerance to self-antigens (29). An example of molecular mimicry is the sequence homology (87%) between the 222–229 region of the major linear B cell epitope of Ro60 kD autoantigen (pep216-232) and Coxsackie virus 2B protein (pepCoxs). In the Stathopoulou study (30), a possible cross-reaction in pSS patients between the antibodies to the Ro60 kD autoantigen and the homologous pepCoxs was suggested. Such a close homology may, on one hand, cause the tolerance for the “aggressor”, but on the other hand, it may lead to the breakdown of self-tolerance and the formation of antibodies that also react with the host’s own antigens (30).

We distinguish a direct structural mimicry relying on the similarity of antigens and causing a reaction against self-antigens (31), a functional mimicry, where different antigens share common surface topologies with respect to a shape or chemical structure, as well as where the flexible paratope accommodates dissimilar antigens by adjusting the structural features according to antigenic epitopes reflecting functional equivalence in the absence of structural correlation (32), and a genetic mimicry, where viral genes are functional and structural homologs of important genes of the immune response (33).

The achieved similarity of the pathogen to the host may elicit an immune response against the antigenic determinants present in both the host and the pathogen. The consequence of the autoimmune response is the activation of autoreactive T or B cells and the destruction of host cells and tissues by the host’s own effector mechanisms, such as the system of phagocytic cells and/or the complement system.

**Table 2** shows examples of the similarity of viral and human antigens and the potential diseases associated therewith.

**Table 2:** Examples of molecular mimicry between viral and human antigens (31–33).

Viral antigen	Human antigen	Associated rheumatic disease or autoimmune disease	Organ/systems affected
Epstein–Barr EBV nuclear antigen (protein)-EBNA	DNA, RNP ribonucleoprotein antigen Ro and La antigens in the nuclear and cytoplasmic ribonucleins	Sjogren syndrome Systemic lupus erythematosus	Skin, mucous and serous membranes, joints, kidneys, central system nervous, blood, exocrine glands
EBV Epstein–Barr nuclear protein 1	Epidermal keratin, collagen type II, actin	Rheumatoid arthritis	joints structures
EBV virus polymerase	Human basic myelin protein	Sclerosis multiplex	Central nervous system
Encephalomyocarditis virus Coxsackievirus B3 (VP1 protein)	Histidyl tRNA synthetase Myosin, tropomyosin, vimentin	Polymyositis myocarditis	Muscles
Epstein–Barr nuclear antigen (EBNA1)	ribonucleoproteins Ro (SSA)	Sjogren’s syndrome	Exocrine glands Epithelium
Viral EBER-1 and EBER-2	Anti SS-B/La antibodies	Sjogren’s syndrome	Exocrine glands (salivary glands) Epithelium

### Bystander activation

Bystander activation is an interesting problem of viruses inducing a non-specific autoimmune response, which results in the creation of an inflammatory environment with cytokine and chemokine production. In susceptible—genetically predisposed—subjects, viruses activate antigen-presenting cells (APC), especially DC, and auto-reactive naïve T cells, which may, in turn, stimulate different immune cells for migration to the infected area (34). During infection, naïve CD8+ cells release tumor necrosis factor (TNF) and IFNγ and produce cytotoxins (perforins and granzymes), in this way inducing apoptosis. Another way leading to the destruction of infected cells is Fas/FasL interaction. FasL protein, which is a type II transmembrane protein that belongs to the TNF family, is expressed on the activated CD8+ T cell surface. Its binding to the Fas receptor (also known as tumor necrosis factor receptor superfamily member 6—TNFRSF6) on the target cell surface is a signal for the caspases’ cascade activation and cell death (34, 35). As a side effect of bystander activation, damage to healthy cells occurs, with a release of self-antigens and the activation of autoimmune reactions. In a healthy, not genetically predisposed, organism, such situation is self-limiting. However, in the case of a dysfunction of the immune system (e.g., immunosuppression, defects of the immune system, and persistent infection), the eradication of immune cells targeting self-antigens—as a prevention of self-antigen antibody production—becomes inefficient.

### Epitope spreading and cryptic antigens

Tissue damage, oxidative stress, and cell death lead to the release of antigens that can become targets for the organism’s own immune system. In inflammation, APC cells can process the released epitopes in such way that, instead of being sequestered, they become immunogenic—leading to the breakdown of self-tolerance and the activity of self-reactive T lymphocytes. This phenomenon is based on the epitope-specific immune response directed against foreign or self-proteins/antigens, which may be spreading to subdominant or even cryptic epitopes of those proteins/antigens (1, 36). In the course of a viral infection, more self-antigens may be released, triggering the *de novo* activation of auto-reactive cells, which also target other epitopes.

### Superantigen theory

The role of superantigens (SAGs) in the mechanism of autoimmunity is controversial. The SAGs are proteins produced mainly by bacteria but may also be produced by virus-infected cells. Superantigens can bind to the TCR receptor regardless of their specificity, causing the activation of T cells (37). Viral superantigens may activate non-specifically to any particular epitope immune response with the activation of a large number of T cells, which produce IFNγ, and with the stimulation of B cells. In the 1990s, the B cell-stimulating SAGs were already discovered, able to activate B cells without cross-talk with T cells. Recently, these mechanisms have been better understood, mainly through the analysis of the influence of bacterial infections (38).

In the context of a discussion about the role of autoimmune processes and immune defense in pSS pathogenesis, the impact of SAG should be considered as a potential source of the direct activation of receptors and a possible factor in the hyperstimulation of the immune system. Although the role of SAGs cannot be unequivocally considered as a source of the autoimmune process, this mechanism of inducing an immune reaction should not be omitted when discussing the role of infections in pSS (39, 40).

The influence of some superantigens (proteins, toxins, etc.) should be taken into account in the search for therapeutic and diagnostic options based on the use of protein engineering methods (41).

**Figure 1:**
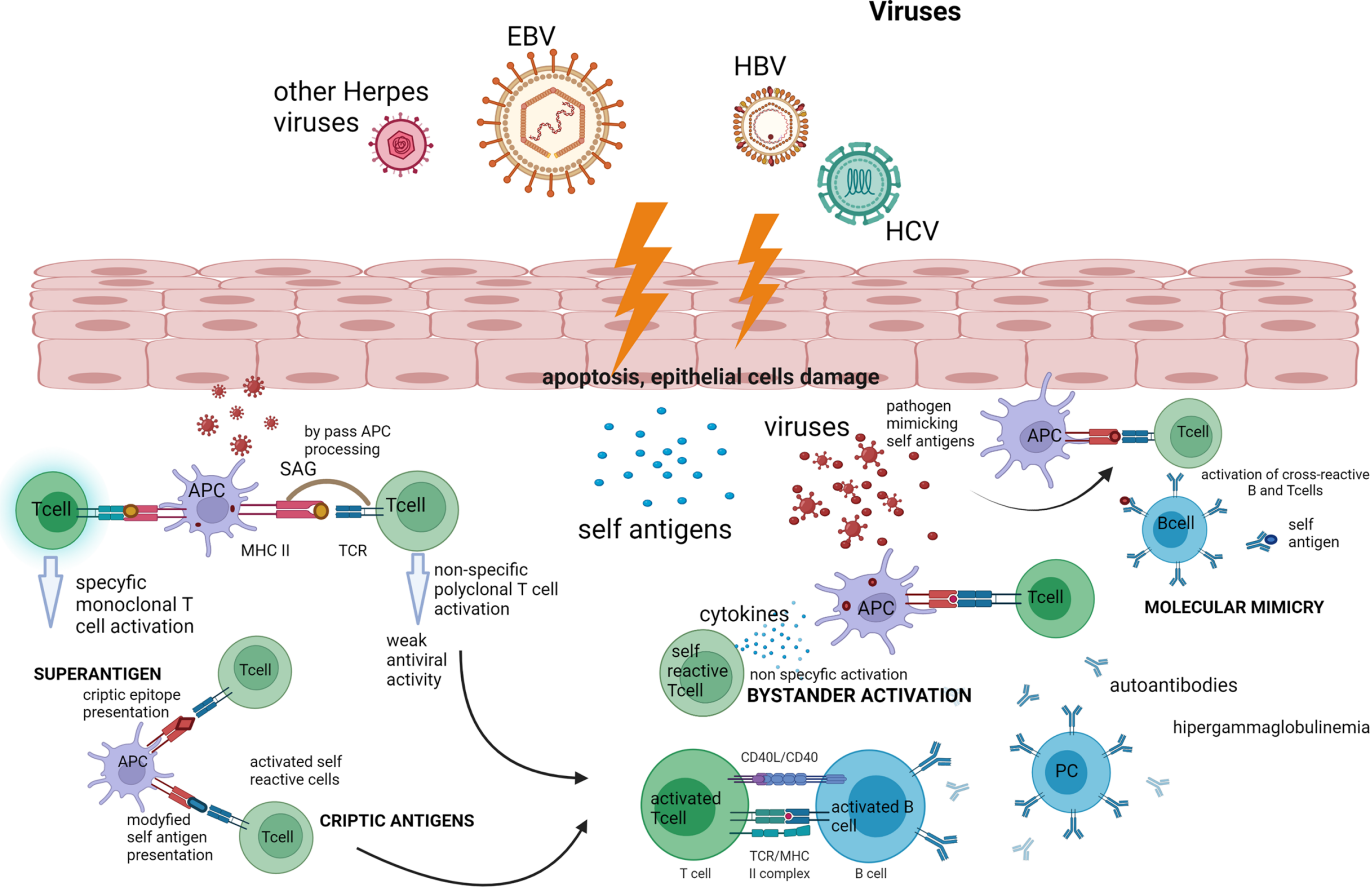
Outline of the possible action of viruses in the pathogenesis of pSS. The presented diagram shows the possible paths of activation of the autoimmune process by viruses such as bystander activation and molecular mimicry and the action of the virus as a superantigen—which are described in full in the text of the article. Created with BioRender.com. APC, antigen-presenting cell; EBV, Epstein–Barr virus; HCV, hepatitis C virus; HBV, hepatitis C virus; PC, plasmatic cell; SAG, superantigen.

In **Figure 1**, an outline of the possible action of viruses in the pathogenesis of pSS is presented.

### Viral cytokines

Viruses can influence the functioning of the host immune system to their advantage by the expression of viral cytokines (virokines), chemokines, and proteins similar in structure to those of the host. Viral cytokines help to create progeny virions and increase the chance of survival of the virus in the host organism (37). Brown et al. (42) first discovered in 1985 that the 140-residue polypeptide encoded by one of the early genes of vaccinia virus (VAV) is closely related to the human epidermal growth factor (EGF) and type I transforming growth factor (TGF). The researchers pointed out that the production of EGF-like growth factor (viral-EGF or vEGF) by virus-infected cells may be the cause of proliferative diseases caused by this family of Poxviruses, such as e.g., rabbit (shope) fibroma virus, Yaba (monkey) tumor virus, and molluscum contagiosum virus. This topic has been explored over the years, especially since the VAV virus belongs to the same family as the smallpox virus. The effect of vEGF inhibition on the migration of infected cells was investigated as a method for preventing the spread of the virus (43). Although a number of viral cytokines, chemokines, receptors, and binding proteins (among others, vEGF, vβ-NGF, vTNFR, and vCXC1) have been identified over the years, their significance for autoimmune diseases and autoimmunity is still debated (37).

Among cytokines attributed to viruses, the role of vIL-10 has been implicated in pSS pathogenesis. As for other cytokines, such as IL-6 or IL-17, involved in the course of viral infections, there is currently insufficient data on the role that their viral equivalents play in the course of pSS.

IL-10 is an immunomodulatory cytokine that inhibits the immune response and the activity of proinflammatory cytokines. The homolog of this cytokine, IL-10- (v), is produced as a result of EBV activity, enabling EBV to escape the host’s response and avoid elimination through inflammation (37, 44). It has been shown that this cytokine can be produced as a result of the activity of other viruses, such as cytomegalovirus. IL-10 (v) sequences are slightly different (two introns) from human IL-10, but IL-10 (v) still possesses immunosuppressive properties (45).

Zhu et al. (46) showed, using a mouse model of Sjogren’s syndrome, the activity of adenovirus-mediated vIL-10 gene transduction (vIL-10 encoded by EBV) in tears and the impact of IL-10 on the clinical features of dry eye syndrome and the lymphocytic infiltration associated with the experimental autoimmune lacrimal gland inflammation.

Viral interleukin-6 (vIL-6) encoded by human herpes virus 8 (HHV8) is only partially identical to the human pro-inflammatory cytokine IL-6 (25% of homology). HHV8 activity is detected, *e*.*g*., in patients with multifocal Castleman’s disease, Kaposi’s sarcoma, and HIV infection (47). There are studies showing the role of IL-6 in pSS, revealing the increased levels of IL-6, IL-17A, and nitrogen oxide in the serum and saliva of pSS patients compared to the healthy control group (48) and the correlation of IL-6 levels with the extent of mononuclear cell infiltration in salivary and lacrimal glands. Fujimura et al. (49) showed that IL-6 induces REG Iα (regenerating gene) transcription in salivary ductal cells through the activation of signal transduction and activation of transcription 3 (STAT3) and binding to the consensus sequence of REG Iα promoter in ductal epithelial cells. The authors concluded that the IL-6/STAT pathway may play a role in the pathogenesis of pSS. Although it can be assumed that viral IL-6 may also potentially play a role in autoimmune diseases, not only in the context of HHV8 infection and Kaposi sarcoma, there are no certain data on vIL-6 in pSS.

The role of the virus homolog of IL-17 (vIL-17) is not yet fully understood. It has been found to be encoded by herpesvirus saimiri (HVS13) and has affinity to T cells. vIL-17 possibly has a beneficial effect on virus survival (50). Interleukin-17 itself is a cytokine that can stimulate T cell proliferation and the secretion of pro-inflammatory cytokine-IL-6, granulocyte colony-stimulating factor, TNF, and chemokines (the chemokine C-X-C motif ligand) such as CXCL1, CXCL2, and CCL20 as well as acute-phase reactants—therefore promoting inflammation (50, 51). Studies in patients with pSS have shown the presence of IL17 and its activating cytokine IL-23 in the lymphocytic infiltrates of the exocrine glands and increased levels of circulating IL17 in the serum and saliva of these patients (52). Knowing the influence of IL-17 on pSS progression and some clinical features of this disease, it can be assumed that vIL-17 may also play a role in both pSS and SS-like disease development.

To sum up, viral cytokine-induced inflammation may promote immune dysregulation or intensify the activity of autoreactive lymphocytes, but there are currently no studies clearly demonstrating the causal role of viral cytokines in autoimmunity.

## The role of Herpesviride

Herpesviruses (HV) constitute a virus family infecting mammals (including humans), birds, reptiles, fish, and oysters. The human herpes viruses are identified in the current taxonomy with symbols numbered from HHV1 to HHV-8; some of them are allocated to subfamilies (α, β, and γ) (45). Humans play the role of a host for herpes simplex virus types 1 and 2 (HSV-1 and 2) and varicella-zoster virus (VZV; HHV-3)—which belong to the α-HV subfamily, cytomegalovirus (CMV; HHV-5), human herpes virus 6 and 7 (HHV-6 and HHV-7)—which belong to β-HV subfamily and Epstein–Barr virus (HHV-4) and Kaposi sarcoma herpes virus (HHV-8)—from the γ-HV subfamily.

Viruses from this family, *e*.*g*., EBV, CMV, or VZV, may stay in a latent phase and reactivate. Although the role of reactivation or increased antibody levels is not explicitly causal for pSS, those phenomena take part in creating immunologic differences between pSS patients and controls (53).

### Epstein–Barr virus

The Epstein–Barr virus (HHV-4) is a DNA virus primarily attacking human B cells, presenting predilection to the salivary glands. It is known that EBV binds to CD21 receptor on the surface of B cells through the viral envelope glycoprotein gp350, while glycoprotein gp42 binds to a human leukocyte antigen as a co-receptor (54, 55). However, other cells without CD21 expression, such as epithelial cells, may also be infected by EBV. Epstein–Barr virus was found in some T cells in T cell lymphoma as well as in epithelial cells in nasopharyngeal and gastric carcinomas (56). It has been shown that, in case of asymptomatic virus carriers and in chronic infection, EBV is mainly confined to B lymphocytes, although it has been suggested that epithelial cells may be the site of EBV replication and amplification rather than the location of the virus in its latent form (57). The establishment of sites of EBV activity is still a subject of research.

It was suggested that, in the pathogenesis of pSS, EBV infection plays an important role in the development of the autoimmune process, which leads to B cell hyperreactivity and may result in the immortalization of B cells. Immortalization is one of the mechanisms that allow EBV to survive in an infected cell and avoid elimination by inhibiting the apoptosis of these cells (58). *In vitro* studies confirmed that peripheral blood resting B cells are transformed by EBV to proliferating B-lymphoblastoid cell lines (LCLs). This is possible thanks to viral proteins, especially EBNA-2. These proteins contribute to the continuous proliferation of B lymphocytes arrested in the lymphoblastoid phase of differentiation and block the transition of the virus to the lytic cycle in most infected cells (4). These LCLs were initially considered as immortal cells that do not become tumorigenic, but it was shown that LCLs are, in fact, mortal (as cells with normal diploid karyotypes and shortening telomeres) (59). Some LCLs, however, become really immortalized because of a strong telomerase activity, aneuploidy, downregulation and mutation of some genes, and modulation of apoptosis. The immortalization may develop into the tumorigenesis of LCLs (60).

Epstein–Barr virus infection status was studied in a large group of patients with multiple sclerosis (MS), which is an immunologically mediated demyelinating disease affecting the central nervous system. Bjornevik et al. (61) investigated 955 subjects (military personnel) diagnosed with MS during their period of service, and 801 MS cases were available to assess EBV infection status; 1,566 controls were also tested. Over the course of 10 years, the researchers collected three samples from each subject for the analysis of EBV infection status, and ultimately only one of the MS-diagnosed subjects remained uninfected, while 35 of them seroconverted from a negative to a positive result before the onset of MS. This study revealed a high rate of seroconversion in the MS group (97%) in comparison with non-MS subjects (57%). There was no confirmation of such a strong association for a similarly transmitted cytomegalovirus (62). Attempts were made to associate an EBV infection with the risk of MS development, but no such association was found. The results of these studies may hold important clues for further research on the effects of EBV on the development of autoimmune diseases.

A persistent EBV infection may cause damage to the salivary glands, resulting in symptoms of dryness. EBV can persist in the human organism in a latent form, allowing the virus to hide in the host cell and reactivate under favorable conditions. In order to survive unnoticed, the virus uses various methods to hide its markers, so that it escapes the control of the host’s immune system. Thanks to the discussed phenomenon of molecular mimicry (see **Table 2**), production of viral IL-10, T cell costimulatory gene CD70 overexpression, or impaired EBV-specific T cell response, EBV modulates host response as well as stimulates autoantibody production. Interestingly, EBV infection can exist in different cells both in the lytic and the latent forms simultaneously (63).

The detection of antibodies against specific virus proteins and the PCR confirmation of the presence of EBV-DNA may be employed by clinicians to establish the status of EBV infection. The more routine methods of determining the infection status include detection of antibodies against nuclear antigen-1 (EBNA-1), early antigen (EA), viral capsid antigen (VCA-IgM and VCA-IgG), noncoding RNA protein (EBERs) and, in certain indications—mainly in patients after transplantation with a suspected post-transplant lymphoproliferative disease (PTLD)—a PCR testing for the presence of EBV-DNA.

Depending on the stage of infection, the lab test results differ. In a recent infection, antibodies against EA, VCA-IgG, and VCA-IgM can be found, and the PCR test for the presence of EBV-DNA is positive. In the case of infection with reactivation, antibodies against EBNA-1, EA, VCA (IgM and IgG classes of immunoglobulin) can be detected. The past infection results in the presence of VCA-IgG (+) with a less probable presence of VCA-IgM (+/-) and EBNA-1 IgG (+/-). In post-transplant lymphoproliferative disease, PCR EBV-DNA is positive and EBER antibodies can be detected, with the presence of other antibodies being less likely (44).

Epstein–Barr virus not only promotes the development of autoimmunity but may also lead to the development of malignancies, especially of lymphoma (55). The higher incidence of EBV reactivation in pSS patients and the expression of HLADR antigens on salivary epithelial cells in this group as well as the increased levels of EBV antigens in infiltrating lymphocytes lead to the conclusion that pSS development is influenced by EBV. Several studies revealed that circulating B cells harboring EBV were present in pSS patients (64). Other evidence came from studies of the saliva of pSS patients, in which it was proved that it activates target genes for AhR (aryl hydrocarbon receptor) and BZLF1 (trans-activator protein—an EBV protein which takes part in switching of the infection phase from latent to lytic). CYP1A1 (the first gene to be transcribed during EBV replication) and Zp130 (synthetic peptide related to ZEBRA—Z Epstein–Barr replication activator) genes were also activated by the pSS saliva (65, 66). A correlation between the levels of anti-La/SSB in the sera and AhR activity in the saliva of SS patients was also found (66).

### Cytomegalovirus

Cytomegalovirus (HHV-5 or CMV) is a common virus (infecting 45–99% of the adult population) usually responsible for mild flu-like or mononucleosis-like symptoms in the general population (67). However, in immunocompromised subjects, it may lead to autoimmune disorders and even the occurrence of severe complications, such as pneumonia, retinitis, hepatitis, meningitis, or gastroenteritis (68, 69).

Following primary exposure, CMV has the ability to establish lifelong presence and latent infection (remains in CD34+ myeloid progenitors). Many cells possess receptors for the virus, one of them being the epidermal growth factor receptor (EGFR), which is important for binding, signaling, and host cell entry (70, 71). The analysis of the effect of CMV in pSS patients is difficult, given its very high prevalence. Infection by CMV emerges as a clinical problem mainly at the time of immunosuppression when CMV may become reactivated, and the course of the infection may be severe. Takizawa et al. (72) presented their work on a large group of patients with rheumatic diseases (RD; *n* = 7,377) who were examined for the development of CMV infection. The infection was confirmed in 151 RD patients, of whom most had a SLE diagnosis (*n* = 74), while only 2.7% (*n* = 4) had a pSS diagnosis. The authors concluded that patients with SLE, dermatomyositis, and microscopic polyangiitis were more prone to CMV disease than patients with other RDs. As the above-mentioned data shows, the role of CMV in the development of pSS itself is not fully understood. The reactivation and the symptoms of CMV infection in pSS patients may be associated with the use of more or less aggressive immunosuppression, as demonstrated by Takizawa et al. in their research (72).

The salivary glands are also a target for CMV, with parotiditis and sialadenitis being described in the course of this viral infection. The saliva of infected subjects is the source of the virus material. Symptoms suggesting pSS may then occur; however, the classification criteria for pSS are not met.

### Varicella-zoster virus

Primary Sjogren’s syndrome as well as other autoimmune diseases are considered as risk factors for the reactivation of varicella-zoster virus and for the occurrence of shingles symptoms in comparison to the general population, which was confirmed in several studies (73). In VZV infection, the varicella-autoantibody syndrome was described (74). The transient presence of antiphospholipid and coagulation protein autoantibodies in VZV infection in children was observed, but the influence of this phenomenon on thrombotic complications was not confirmed.

In the study of Chakarvarty et al. (75), the odds ratio for VZV infection was 1.03 (95% CI: 1.02–1.05) for each year of age, and the risk of VZV infection was at 1.8 (95% CI: 1.2–2.8) for pSS and at 2.7 (1.7–4.3) for SLE patients. In the group of SLE patients, Ro positivity was predictive of an earlier onset of VZV, which, however, was not observed in the group of pSS patients. The authors concluded that, in pSS, there is an increased risk of reactivation of VZV infection compared to healthy individuals but less frequently than in SLE or in the group of elderly patients. Anti-Ro seropositivity, but not a concentration of the studied cytokines (bLyS, IFN alpha and gamma, IP-10, CxCL-13, E-selectin, and MIP-1 beta), was associated with the development of VZV infection in both groups.

### Roseoloviruses

Roseoloviruses also belong to the Herpesviride family and affect more than 90% of the general population. This data applies to the seropositivity or latent infection phase, as a majority of affected subjects are asymptomatic (76). Roseoloviruses are lymphotropic beta-herpes viruses. Human roseoloviruses include human herpes virus-6 (HHV-6A and HHV-6B) and human herpes virus- 7 (HHV-7). The variant of HHV-6 (HHV-B) is more frequent than HHV-7. These viruses mostly affect children and cause known diseases such as febrile infant infections, sometimes with rash (roseola infantum). Infections with those viruses are self-limiting, but viruses can stay in both active and latent forms. The infection can persist for a whole life. Viruses may replicate in peripheral blood mononuclear cells. These viruses may also locate in the salivary glands, and viral DNA can be detected in the saliva by the use of PCR (77). It has been proven that the reactivation of an infection with roseoloviruses may occur in an immunocompromised person. The link between central nervous system diseases such as multiple sclerosis and encephalitis leads to this genus of viruses being intensively studied (78, 79).

Ranger-Rogez et al. (80) indicated in their study higher titers of HHV-6 antibodies in the group of pSS patients than in healthy subjects. The study on RA patients with or without SS revealed that, in both groups, there was a significantly increased frequency of latent viral infection (sevenfold higher, *p* = 0.018 for HHV-6) compared to normal controls—interestingly only in cells isolated from saliva (81). More up-to-date research has been done on SLE, concluding that HHV-6 infection may contribute to the development of this autoimmune disease, but it is also noted that the autoimmune disease may cause the reactivation of human roseoloviruses (82). The research performed on animal models, mainly non-primates, e.g., macaques or marmosets, tried to establish the relationship between the development of AIDS or neurological diseases and an infection with roseoloviruses. Currently, mouse models, such as mice, naturally resistant to herpes virus infection or transgenic mouse models will allow for a better understanding of the role of HHV-6 infection in humans (83). In the work of Bigley et al. (84), it has been shown in a mouse model that a neonatal infection with murine roseolovirus related to human roseoloviruses causes autoimmune gastritis in adult subjects, the production of a number of autoantibodies, and an increase of thymocyte apoptosis at the negative selection stage. This is the first study which provides direct evidence that a roseolovirus infection can induce autoimmunity and the production of pSS-associated autoantibodies (anti-SSA/Ro and anti-SSB/La).

Undoubtedly, this topic has not been exhausted, and the role of roseoloviruses in pSS should be re-investigated and described in more detail.

### Retroviruses

Viruses, which were later assigned to the Retroviridae family, have been the subject of research since the beginning of the 20th century. In the 1970s, the attention of clinicians in the southern islands of Japan and in the USA was drawn to the emergence of numerous leukemia cases from mature T lymphocytes, and research led to the isolation of the human T cell leukemia virus (HTLV-I) (85, 86). In 1982, another virus which causes hairy cell leukemia—HTLV-II with 70% genomic homology to HTLV-1—was identified (87). The discovery of the viral reverse transcriptase (RTs) which has DNA polymerase and rnase H activity (88) was crucial for the understanding of the mechanisms of replication of retroviruses. In 1983, human immunodeficiency virus (HIV) was isolated in 1983 by a group of researchers under the direction of Luc Montagnier (89), who based their work on the previous research of Robert C. Gallo on HTLV-1 (90).

### Human T lymphotropic virus type 1

Although the prevalence of HTLV-1 virus in some parts of the world is high, only about 5% of people develop symptomatic infection (91). Adult T cell leukemia (ATL) and HTLV-1-associated myelopathy (HAM) have been directly associated with HTLV-1 infection.

Studies conducted on transgenic mice have proved that subjects infected with retroviruses develop autoimmune diseases such as Sjögren’s syndrome, polymyositis, or rheumatoid arthritis more often than the general population (92). The HTLV-1 virus equipped with the tax and bZIP gene (HBZ) infects salivary gland cells, leading to the increase of the concentration levels of inflammatory factors, e.g., ICAM-1, IP-10-, and chemokines, such as RANTES (regulated on activation, normal T cell expressed and secreted) (93). In the course of HTLV-I infection, B cells are inhibited, and the production of autoreactive antibodies is reduced (94).

Greene at al (95). confirmed the involvement of the lacrimal and salivary glands resembling Sjögren’s syndrome in HTLV-1 tax transgenic mice. Mariette et al. (96) also described the presence of the tax retrovirus HTLV-1 gene in the salivary gland cells of patients with pSS. Nakamura et al. (97) noticed that, in patients with pSS and HAM, the titer of SSA/Ro antibodies was significantly lower compared to patients with pSS without HTLV-1 infection despite the lacrimal gland involvement being more severe in this group. An interesting observation was that the degree of mononuclear cell infiltration, assessed as focus score in the biopsy of the MSGB in both groups, was similar (98).

Terada et al. (99) pointed out that, among seropositive patients with pSS, antibodies against HTLV-1 in the IgA class are commonly found in saliva. In a subsequent study, the authors noted that, in HTLV-1-positive patients with pSS, there is a lesser destruction of the salivary glands than in HTLV-1-negative patients (94). Infection with HTLV-1 may inhibit cell apoptosis and enhance proliferating signals, which may explain the less intense destruction (100).

Magnetic resonance imaging of salivary glands in the group of patients with HTLV-1-associated myelopathy did not show typical changes for pSS, but the salivary flow rate was similar in both groups (101). In Brazil, 129 HTLV-1-seropositive patients were screened for meeting the criteria for pSS. Many of them had clinical symptoms of eye and mouth dryness (46 cases of dry mouth, 18 dry eyes, eight with confirmed decreased saliva secretion, and two with salivation disorders), but only one patient had antibodies characteristic for pSS (102). A biopsy of the minor salivary glands was performed in six patients of that group, revealing infiltrates with mononuclear cells characteristic for pSS (103). Analyzing the above-mentioned data, it seems that the HTLV-1 virus can act in two ways: in some patients, it stimulates an autoreactive response and causes the development of full-blown pSS, but it can also induce a non-specific inflammation of the salivary glands as manifested by excessive dryness. Moreover, in HTLV-1-positive patients, a smaller number of ectopic GC and a low expression of CXCL13 in mononuclear cells infiltrating the glands are observed compared to pSS patients without HTLV-1 infection (98).

Similar with other retroviruses, HTLV-1 primarily has an affinity to TCD4 + lymphocytes but can also infect other cells, e.g., salivary duct epithelial cells, which attract T lymphocytes in patients with pSS by secreting interferon-induced 10-kD protein (CXCL10) and monokines (CXCL9) (103). It is suggested that the change of the inflammatory environment in the epithelial cells of the salivary gland ducts and the significant transmigration potential of CD4+ T cells infected with HTLV-1 retrovirus may lead to the development of pSS in these patients. Recent studies have also focused on the expression of TCR3 receptor binding to viral RNA on salivary gland epithelial cells (103). The relationship between pSS and HTLV-1 may be further illustrated by studies on the role of HTLV-1 basic leucine zipper (HBZ) gene expression. An increased expression of this gene and induction of the chronic inflammatory process through Foxp3 were shown in the salivary gland tissue of patients with both ATL and Sjögren’s syndrome. This may indicate the connection of these diseases (103).

In summary, it seems that HTLV-1 virus, by infecting salivary gland epithelial cells, changes their cellular functions, which may induce the development of pSS as demonstrated by Nakamura et al. (103). Lee et al. (104) confirmed HTLV-1 presence in the labial salivary glands of patients with pSS and suggested that it can be a special clinical subgroup.

### Human immunodeficiency virus

The human immunodeficiency virus has an affinity to CD4+ cells but also affects macrophages and dendritic cells (105). The viral envelope glycoprotein gp120 is recognized by CD4+ molecule, which has a greater affinity for it than for its dedicated ligand, the MHCII molecule. The virus combines its genetic material with the material of the cell and goes into the latent phase. Even in the latent state, it maintains a high level of replication in the lymph nodes and lymphoid cells of the digestive system (106).

Subsequent studies have tried to explain the mechanisms of autoimmune disorders caused by HIV. The destruction of CD4+ cells and the activation of autoantigens and cytotoxic cells were taken into account. The phenomenon of molecular mimicry with the gp41 antigen and polyclonal activation of B lymphocytes has also been described (107). For many years, it has been pointed out that, in a population of HIV-positive patients, there is an increased frequency of symptoms that may correspond to pSS. In the study by Kordosis et al. (108) published in the 1990s, it was noted that, among 77 HIV-positive patients, 26 had symptoms of dry eyes and/or mouth. Furthermore, 14 patients from this group had MSGB performed, among which six had monocellular infiltrates meeting the criteria of focus score in pSS and four had mucoid degeneration of the stroma. However, in the immunohistochemistry assessment, CD8+ cells dominated in the infiltrate in contrast to pSS, where mainly CD4+ are observed. Interestingly, none of the studied patients had anti-SSA/Ro or SSB/La antibodies, but all of them had hypergammaglobulinemia (108). Many years of observations have led to the identification of a disease entity in HIV-infected patients called diffuse infiltrative lymphocytosis syndrome (DILS) with a clinical picture similar to Sjögren’s syndrome. The criteria of DILS diagnosis, established in 1995, are as follows: a confirmation of HIV infection (positive serology), bilateral salivary gland enlargement or xerostomia, persistence of symptoms for 6 months or more, and histologic confirmation of salivary or lacrimal gland lymphocytic infiltration without granulomatosis or neoplastic involvement (fulfillment of all criteria is required to make a diagnosis) (109). In the course of DILS development, symptoms such as bilateral painless parotid and lachrymal gland enlargement—sometimes even with severe sicca symptoms—as well as lymphocytic interstitial pneumonitis, hepatitis, myositis, lymphadenopathy, polyneuropathy, and aseptic meningitis may occur. The main immunological feature of DILS is the proliferation of CD8+ T cells, with infiltrations containing these cells affecting multiple organs. DILS is suggested to reflect an over-response to HIV by the host. During HIV infection, marginal zone B cell MALT lymphomas were described, which also resemble pSS and confirm the possibility of a chronic stimulation of these cells by an infection, leading to the loss of control of multiplication and to lymphoproliferation. It was also shown that subjects with dryness syndrome and HIV have higher serum levels of interferon gamma (110).

Interferon gamma (IFNγ) plays an important role in antiviral defense. Its production clearly grows in the early and acute phase of HIV infection, and in the later stages of the disease, IFNγ, together with other inflammatory cytokines, is involved in persistent immune activation (111). The activity of IFNγ exacerbates the effects of HIV and plays a role in AIDS development. Interferon gamma and type I IFNs may also promote the IFN signature by the upregulation of IFN response genes (IRGs) in pSS (111).

HIV infection can be seen mainly as an element in the differential diagnosis of Sjogren’s syndrome—especially now, thanks to highly active antiretroviral therapy. As HIV infection has become a chronic disease and its mortality has significantly decreased, it can be encountered more often in medical practice. The prevalence of DILS currently is significantly reduced (110).

## The hepatitis viruses

### Hepatitis C virus

Hepatitis C virus is a small single-stranded RNA virus, a member of the hepacivirus genus in the Flaviviridae family. This virus is a recognized cause of hepatitis, a risk factor for cirrhosis, liver tumor, and other cancers as well as lymphoma development. The hepatitis C virus presents not only tropism to hepatic cells but also sialo- and lymphotropism (112). For decades, there was an ongoing discussion on whether HCV infection is a cause of pSS or only a mimicker of this disease due to the symptoms of dryness, arthralgias, and other symptoms similar to the clinical picture of pSS (113). Hepatitis C virus infection, especially the chronic one, is associated with extrahepatic disease manifestations, stimulation of B-cell activity by BAFF, and autoimmune phenomena, including emerging cryoglobulins and immune complexes, leading to vasculitis, polyneuropathy, lymphoproliferation, and, as recently suggested, endocrine manifestations such as thyroid autoimmunity or diabetes mellitus type 2. Among autoantibodies, ANA and RFs are quite common, but others, e.g., anti-GM1 ganglioside and anti-sulfatide, may also be observed (114). Virus *per se*, especially in a chronic infection, may be a cause of focal sialoadenitis, with usual mild dryness symptoms (even in 50% cases) and inflammatory infiltration of mononuclear cells in the histopathological assessment being observed (115).

In the context of the impact of HCV on the development of pSS, the available meta-analyses and individual studies focus on the population of patients with the presence of HCV-RNA and HCV antibodies, and the authors indicate an SS-like disease more often than pSS (113). In both diseases, pSS and chronic HCV infection, there is an increased risk of lymphoma (especially B cell lymphomas) development with the predominance of changes in the target organs—salivary glands in pSS and salivary glands and liver in HCV (114). Dryness symptoms and sialadenitis associated with hepatitis C infection are also linked to the increased risk of lymphoma development similar to that in pSS (115). To conclude, the immunological activity of HCV virus, its tropism to the salivary glands, and the possibility of induction of a pSS-like disease hinder the diagnostic process of patients with suspected pSS. The influence of a previous infection of HCV on the development of pSS has been discussed for years; it has even been suggested to be treated as a subtype of pSS (113). Eventually, HCV viremia and active infection were established as the exclusion criteria in pSS diagnosis.

An active HCV infection may be a multisymptomatic and multisystem disease with chronic fatigue, arthralgia, general symptoms (such as fever, weight loss, and loss of appetite), skin changes, such as Raynaud’s phenomenon, livedo reticularis, or purpura (the latter most often associated with vasculitis in the course of cryoglobulinemia). Symptoms related to the gastrointestinal tract, resulting directly from the damage to the hepatic cells, do not have to be dominant or evidently present at the beginning of an HCV infection. The involvement of the salivary glands, with their enlargement and dryness, may also be a symptom of an HCV infection due to the predilection of this virus to lachrymal and salivary glands epithelial cells—which was confirmed by the detection of HCV-RNA in infected patients’ saliva. In the course of HCV, dryness is usually mild. Ramos-Casals (116) proposed the term “SS secondary to HCV”, but it seems inadequate, as in the case of HCV we are dealing with a wide range of phenomena, which depend on the stage of the infection (active, persistent, or past infection) and vary from pSS-like symptoms mimicking or accompanying pSS to the stimulation of the immune process, which may, in fact, play a role in pSS development.

### Dengue virus

According to Chang et al. (117), an infection with a dengue virus (DV), another virus from the Flaviviridae family, is lowering the risk of pSS development. The authors presented results demonstrating that the incidence rate of pSS was lower in the DV cohort than in the non-DV cohort (0.51 vs. 1.47), with HR of 0.30 (95% CI: 0.13– 0.67), matching age, gender, and residence (117).

### Hepatitis B virus

The role of HBV infection in the pathogenesis of Sjogren’s syndrome seems quite unclear. In Spain, Marcos et al. (118) conducted studies on a large population of patients with Sjögren’s syndrome. The study focused on the prevalence of HBV infection in this population and the clinical and laboratory evaluation of these patients. The presence of HBsAg was detected in five out of 603 patients with Sjogren’s syndrome. Hepatitis B virus and HCV co-infection were not found. The main symptoms observed in the group with HBV infection were dryness and arthralgia or arthritis; the dominant positive laboratory tests were RF and ANA. However, cryoglobulinemia was not observed in contrast to HCV infection and pSS, where the presence of cryoglobulins is not rare. The authors stated in conclusion that the incidence of HBV infection among patients with pSS was found to be similar with that of the general population.

Similar research in Taiwan was carried out by Chen et al. (119), and their observations also confirmed higher RF concentrations in patients with the presence of HBsAg. Additionally, attention was drawn to a lower lung involvement rate in comparison with other pSS patients (119).

Another study conducted with a large group of pSS patients, including over 9,500 subjects, assessed the prevalence of hepatitis B and C infections in this group. It was estimated that, in the group with chronic HBV infection, the risk of developing Sjogren’s syndrome is much lower (OR 1.25, 95%CI = 0.95–1.24) than in the case of HCV infection, which is associated with the risk of SS (OR = 2.49, 95% CI = 2.16–2.86) (120).

Ram et al. (120) suggested that HBV infection may even play a protective role in relation w autoimmune diseases. In another study, Ramos-Casals et al. (121) investigated the frequency and symptoms of liver involvement in 475 patients with pSS. Among 129 patients with liver involvement, only one had a confirmed HBV infection. The presence of HCV in the cited study was detected much more often, as in other studies (121).

For years, there was a high prevalence of HBV infection in Taiwan, and about 20% of the general population had HBV seroprevalence (the differences were mainly due to age and gender). Therefore, Taiwan became particularly interesting for studying the influence of HBV on the development of other diseases, including autoimmune diseases (122). The compulsory vaccination program has certainly improved the situation in the last 30 years (since 1984). Tung et al. (123) identified 26,147 adults diagnosed with HBV infection and chose 3,268 patients who, at some point, received nucleotide therapy (treated group) and compared them with the second untreated group (*n* = 13,072) in terms of pSS development. The authors found that the risk of pSS development was significantly lower in the treated group [15-year cumulative incidence, 2.4%; 95% confidence interval (CI), 1.4–3.7%] than in the second group (7.1%; 95% CI, 2.5–15.2%) (*p* = 0.015). The authors concluded that the obtained data points to the positive impact of the treatment of HBV infection with nucleotides on lowering the risk of Sjögren’s syndrome development, which reversely may indicate some role of this viral infection in the pSS pathogenesis (123).

Although there are reports that HBV infection may be a protective factor against the development of SLE, MS, and DB1 (statistical significance in comparison with healthy controls, *p* < 0.05), there is no sufficient evidence to establish the same for pSS (no statistical significance, *n* = 82, 6.1 vs. 10.7%, healthy controls) (120). The authors argue that a low percentage of antibodies to the core antigen (HBcAg) in patients with SLE, MS, and T1D in comparison to healthy controls may suggest that the infection with some viruses, in this case HBV, may have a protective effect (120).

### Hepatitis delta virus

Interesting observations were presented by Weller M et al. (124), who showed an increased presence of hepatitis delta virus (HDV) in 50% of the assessed pSS patients. The presence of the HDV antigen was confirmed in the minor salivary gland tissues. Significantly, patients with the presence of HDV in the minor salivary glands did not have detectable hepatitis B virus surface antigen (HBsAg) or antibodies to HBV or HDV. This is interesting, taking into account that HDV is strongly associated with HBV and depends on this virus in terms of release, replication, and transmission. The presence of HDV may be a prognostic factor for the course of this dual infection (125). In the presented results, the expression of HDV antigens was associated with a decrease in stimulated salivary flow, the production of autoantibodies, and the intensification of focal lymphocytic infiltrates in the tissue of minor salivary glands (124).

### Hepatitis G virus

The genome of this RNA virus is similar in its organization to HCV virus; the sexual transmission of this virus was suggested and confirmed by some studies on homosexuals, sex workers, and homosexual patients with a coexisting HIV infection (126, 127). This virus infects peripheral blood mononuclear cells, mainly in B and T cells and the bone marrow. The primary replication of HGV in hepatocytes is under discussion as HGV hepatitis usually (75%) proceeds without an increase in liver enzymes (128)

In 1998, Font et al. (129) presented the results of a study on 100 pSS Spanish patients and concluded that, in this population, the prevalence of HGV infection is low (seroprevalence confirmed only 4 versus 3% in blood donor group). In this study, patients with confirmed HGV did not differ from those without infection; in one case, the increase in liver enzymes was most likely due to the coexistence of HCV infection (129). It seems that this virus does not significantly contribute to the development of autoimmune diseases, including pSS, and its co-presence with other viruses may even be a protective factor, as has been noticed in the case of HBV.

### Hepatitis E virus

This is an RNA virus not only with hepatotropism but also with extrahepatic manifestations, e.g., in the intestine, kidneys, neurons, and lymph nodes. The seroprevalence of hepatitis E virus (HEV) IgG depends on the geographical region, the assay used, and the study cohort (age, ethnicity, and eating habits, especially a swine meat diet). Infection with this virus is rarely studied and detected in Europe, which may lead to its underestimation. Hartl et al. (130) showed the high anti-HEV IgG seroprevalence in the south of France and the lowest level in Scotland. Some of those infected may not have symptoms or may have mild transient unspecific symptoms, e.g., fatigue, nausea, itching, rare jaundice, and changes in laboratory test, such as elevation of liver enzymes. Some data suggests that the infection with a genotype 3 (GT3) of this virus is associated with extrahepatic manifestations and autoimmune diseases (131). Replication of this virus in the PBMC was confirmed in the acute HEV infection (132). During the HEV infection, a mix of cryoglobulinemia and cryoglobulinemic vasculitis may develop similarly as in the course of HCV. Fraticelli et al. (133) described the case of a pSS patient with cryoglobulinemic vasculitis and nervous system involvement treated with rituximab and mycophenolate mofetil, who developed symptoms of hepatitis. The authors described successive hypotheses concerning the cause of the active hepatitis, including autoimmune hepatitis, and the associated treatment modifications, up to taking into account the HEV infection and, after confirming this, the application of a targeted therapy (133). The conclusion that was drawn from this is that an unexplained hepatitis in immunosuppressed patients treated for systemic autoimmune diseases should be investigated for a possible HEV infection (132). In the current literature, there are studies describing the association of HEV with mixed cryoglobulinemia (134, 135), which may also be important in the context of pSS, but at the moment there are no broader studies on its relation to pSS itself.

### Coxsackie

Coxsackie virus is a single-stranded RNA virus which belongs to the Picornaviridae family. Coxsackie viruses are divided into two main groups: Coxsackie A and B. Diseases caused by Coxsackie A are quite common in childhood with mild symptoms or an asymptomatic course such as, e.g., hand, foot, and mouth disease or herpangina. Different serotypes of Coxsackie B viruses can cause muscle, heart, and nervous system infections.

The B4 Coxsackie viruses (CB4) and C1 (CB1) serotypes are being researched as potential risk factors for developing autoimmune diseases. Their role in diabetes mellitus type 1, by showing their destructive activity on beta cells on Langerhans cells and pancreas islets, has been confirmed (136). Triantafyllopoulou et al. (137) demonstrated a relationship between Coxsackie virus infection and pSS, presenting the evidence of minor salivary gland cell infection (due to the presence of main antigenic capsid protein VP1) in pSS patients and not finding such an infection in patients with secondary SS, controls with other rheumatic diseases without secondary SS and healthy volunteers. However, these data should be carefully analyzed due to the small number of the study groups (pSS: *n* = 12, sSS: *n* = 13, control: *n* = 14 with rheumatic diseases, and *n* = 2 healthy volunteers). A cross-reaction between antibodies to the major epitope of Ro60 kD autoantigen and a homologous peptide of Coxsackie virus 2B protein, as mentioned previously, was also shown (30). Gottenberg et al. (138), however, did not confirm the relationship between the infection with Coxsackie virus and Sjogren’s syndrome by examining the presence of viral material in MSGB. It should be noted that this study was carried out on a small group of patients with pSS.

### Parvovirus B-19

Due to the high seroprevalence of parvovirus B19 (p19V) IgG antibodies in the general population, estimated as 40–60% in children and adult population (even higher in elderly people—up to 75–85%), it was also taken into account as a potential environmental factor influencing the pathogenesis of pSS and other autoimmune diseases (139, 140). This virus shows tropism to human bone marrow.

De Stefano et al. (141) studied the presence of p19V DNA in the MSGB of patients with pSS and in healthy volunteers (all participants agreed to have MSGB, including the healthy controls). At the same time, the status of antibodies to p19V was assessed in these two groups. B19DNA was found in the smaller salivary glands in both groups; there was no association between the presence of viral DNA and the intensity of infiltration in pSS patients. Therefore, no relationship between pB19 infection and pSS was demonstrated, and the presence of viral genetic material was considered random.

By contrast, Ramos-Casals et al. (142) focused on the clinical significance and the immune picture of patients with pSS and pB19 parvoviral infection. Eighty patients diagnosed with pSS were examined for the presence of antibodies and genetic material of this virus. In 35% of them, a past infection with parvovirus was confirmed, and none had an active viremia. An interesting observation was the more frequent finding of leukopenia and thrombocytopenia in past p19V-infected patients in this group (142). Thrombocytopenia and leukopenia occurring during B19 infection are linked to the cytotoxicity of the parvovirus B19, which is directly related to the cytotoxicity of its non-structural protein (NS1) (143, 144). Several studies from the 1980s confirmed the effect of NS1 on bone marrow cells (144–146). This protein is also associated with arthritis and persistent B19 infection, which was also proved in animal models (146). Parvovirus infection was also assessed as a factor influencing lymphoproliferative processes in patients with Sjögren’s syndrome. Ultimately, however, no such direct relationship was proven (147).

Based on the observations so far, it seems that pB19 does not directly affect the pathogenesis of pSS despite its importance in RA or SLE.

### SARS CoV-2 virus

At present, no broader conclusions can be drawn about the risk of developing pSS after a SARS-CoV-2 infection, although it is known that xerostomia may also be a prodromal symptom of COVID-19. This clinical observation applies to patients with acute infection of a limited duration time (148). In the coming years, it will probably be possible to assess to what extent the SARS-CoV-2 virus may affect the development of pSS or cause symptoms similar to this autoimmune disease. An interesting multicenter observation revealed that, among pSS patients affected with SARS-CoV-2 virus, 57% remain symptomatic after 5 months. It was estimated that the risk of post-COVID-19 syndrome in hospitalized pSS patients is eight times higher than in non-hospitalized patients. The increased CRP levels and the use of hydroxychloroquine in the treatment of pSS were considered as risk factors for post-COVID-19 syndrome in this group (148).

In **Table 3**, the main viruses with their targeted cells and main diseases with which they are associated are presented. Whereas **Table 4** compares the clinical features of pSS and HCV, HIV and CMV infection.

**Table 3:** Main viruses involved in the pathogenesis of pSS.

Virus	Genetic material	Targeted cells	Circumstances of activity and main diseases
EBV	DNA	B cells	Sialadenitis Reactivation in immunocompromised subjects—PTLD, hepatitis Mononucleosis Nasopharyngeal cancer Burkitt’s lymphoma Oral hairy leukoplakia Hodgkin disease (non-related and related to HIV)
CMV	DNA	Epithelial cells, endothelial cells, fibroblasts, and smooth muscle cells	Mononucleosis-like illness Severe complications in immunocompromised subjects including patients with AIDS: CMV-organ disease (retinitis, esophagitis, colitis, pneumonitis, hepatitis)
HTLV-1	RNA	T cells	Adult T cell leukemia, HTLV-1-associated myelopathy
HIV	RNA	T cells, macrophages, and dendritic cells	Flu-like symptoms (acute infection) asymptomatic stage (chronic infection) AIDS with opportunistic infections
HCV	RNA	Hepatocytes, dendritic cells, and B cells	Sialadenitis, chronic hepatitis, cirrhosis, hepatocellular carcinoma, extrahepatic manifestations (organs and systems involvement)
HBV	DNA	Hepatocytes	Acute and chronic hepatitis, cirrhosis, and hepatocellular carcinoma, Acute necrotizing vasculitis—polyarteritis nodosa (circulating immune complexes)* Other extrahepatic manifestations (organ and system involvement)
pB19	DNA	EPCs	In children: erythema infectiosum, thrombocytopenia, ITP In adults: arthralgia, arthritis, anemia, neutropenia, thrombocytopenia

AIDS, acquired immune deficiency syndrome; EBV, Epstein–Barr virus; EPCs, erythroid progenitor cells; CMV, cytomegalovirus; ITP, idiopathic thrombocytopenic purpura; HBV, hepatitis B virus; HCV, hepatitis C virus; HIV, human immunodeficiency virus; HTLV, human T-lymphotropic virus; pB19, parvovirus-19; PTLD, post-transplant lymphoproliferative disease.

**Table 4:** Differences between pSS, HCV, HIV and CMV (3, 7, 77, 85, 124, 126, 149, 150).

	Sjögren’s syndrome	HCV	HIV	CMV
Age	40–50	All	All/young adults	Young/adults
Gender	F>M	All	M>F	Heterosexual transmission F>M Homosexual transmission M>F
	Dryness mild to severe	Dryness usually mild, medium	Dryness even severe (DILS)	Mouth dryness Possibility of HIV coinfections
Antibodies	Anti-SSA/Ro, anti SSB/La ANAs	Anti-HCV Ab presence of cryoglobulins; presence of other autoantibodies is rare, but when Abs are present, they are usually at low levels; ANAs are in low titers	Anti-HIV Ab; presence of other autoantibodies is rare, but when Abs are present, they are usually at low levels; ANAs are in low titers	Anti-CMV Anti-LKM (autoimmune hepatitis) ANAs may be present in low titer
RF IgM	May be highly positive ++/+++	+	+	+
Histopathology	Mainly CD4+	CD4+, CD20+	CD 8+	CD 34+,
Target cells	B cells	Hepatocytes, dendritic cells, and B cells	CD4+	Epithelial cells, endothelial cells, fibroblasts, and smooth muscle cells
Target organ	Exocrine glands Extraglandular organ and system involvement	Liver, salivary glands	Lymphoid tissue	Lymph nodes, central nervous system, liver, bone marrow (coagulopathy, hemophagocytosis)
Organ/system involvement	Pulmonary, nervous system, kidneys, gastrointestinal tract (liver)	Gastrointestinal tract, liver	Musculoskeletal, nervous system, pulmonary, gastrointestinal, skin	Upper respiratory tract, pulmonary, gastrointestinal (liver, spleen)
Possible connection with oncology	MZBL (marginal zone B cell lymphoma); MALT	Hepatocellular carcinoma	Kaposi Lymphoma Others depending on opportunistic infection HPV—cervical, anal, oropharyngeal, penile, vaginal, and vulvar cancer EBV—non-Hodgkin and Hodgkin lymphoma HBV/HCV—hepatocellular carcinoma	Breast and gastric cancer Glioblastoma

HPV, human papilloma virus; EBV, Epstein–Barr virus; HBV, hepatitis B virus; HCV, hepatitis C virus.

The effectiveness of anti-viral therapies in the treatment of autoimmune diseases is also being debated. Dreyfuss (151) suggests that drugs such as acyclovir (inhibitor of herpes virus DNA polymerase) or raltegravir (inhibitor of the activity of HIV-1 integrase), which act by inhibiting the activity of the virus, may also have a positive effect on the inhibition of autoimmune diseases such as RA, SLE, or MS. An example of a research regarding this problem is a work by Friedman et al. (152) lovir in patients with MS, although no statistically significant relationship has been proven between such treatment and the clinical improvement or changes in the MRI scans of patients. There are also analyses showing the benefits of anti-viral therapies in autoimmune diseases. Further interesting results were presented by Bech et al. (153) in the study of the efficacy of valacyclovir in patients with MS. Although they did not show improvement in the whole group with relapsing–remitting MS, a reduction in the number of relapses and a decrease in active changes in MRI were observed in the subgroup with very high disease activity. There are descriptions of positive therapeutic effects of anti-viral therapy (zidovudine) in combination with immunosuppressive drugs in the treatment of myelopathy in patients with HTLV-1 infection and Sjogren’s syndrome confirmation (154).

The above-described examples show the potential usefulness of anti-viral agents in autoimmune diseases; however, this topic requires a separate extensive study reaching beyond the scope of this article.

## Conclusions

The role of viral infections in Sjogren’s syndrome is a subject of ongoing research, but currently it is possible to demonstrate their impact on pSS with a high degree of certainty only in the case of some of the investigated viruses.

The Epstein–Barr virus is suggested as the one most involved in the pathogenesis of pSS. The HCV virus plays a dual role in pSS—on one hand acting as a factor promoting the pSS development (past infection) and on the other as an active HCV infection that constitutes an exclusion criterion for the diagnosis of pSS.

At present, the knowledge of the role of retroviruses such as HTLV-1 in the development of pSS is also increasing. The SARS-CoV-2 pandemic may lead to the expansion of our knowledge on the impact that viruses exert on the development of autoimmune diseases, including pSS.

## Author contributions

MM: concept of the work, literature collection, writing, and acceptance of the final manuscript. KK-G: literature collection, writing, and acceptance of the final manuscript. All authors contributed to the article and approved the submitted version.

## Conflict of interest

The authors declare that the research was conducted in the absence of any commercial or financial relationships that could be construed as a potential conflict of interest.

## Publisher’s note

All claims expressed in this article are solely those of the authors and do not necessarily represent those of their affiliated organizations, or those of the publisher, the editors and the reviewers. Any product that may be evaluated in this article, or claim that may be made by its manufacturer, is not guaranteed or endorsed by the publisher.

## Abbreviations

APRIL, a proliferation-inducing ligand; BAFF, B cell-activating factor; CMV, cytomegalovirus; C-X-C motif, chemokine; CXCL, ligand; EBV, Epstein–Barr virus; GCs, germinal centers; HHV, human herpes virus; HIV, human immunodeficiency virus; HLA, human leukocyte antigen; HTLV, human T-lymphotropic virus; IgG-RD, IgG4 related disease; IRF5, interferon regulatory factor 5; IRGs, interferon response genes; IL, interleukin; MSGB, minor salivary gland biopsy; pB19, parvovirus B-19; RA, rheumatoid arthritis; RF, rheumatoid factor; STAT4, signal transducer activator of transcription 4; SAGs, superantigens; SLE, systemic lupus erythematosus; TRIM, tripartite motif; TNF, tumor necrosis factor.
